# Integrative Bioinformatics Analysis Reveals Potential Gene Biomarkers and Analysis of Function in Human Degenerative Disc Annulus Fibrosus Cells

**DOI:** 10.1155/2019/9890279

**Published:** 2019-02-21

**Authors:** Yin Xunlu, Feng Minshan, Zhu Liguo, Zhan Jiawen, Wei Xu, Yu Jie, Wang Shangquan, Yin He, Liang Long, Han Tao, Li Xuepeng

**Affiliations:** The No. 2 Spine Department, Wangjing Hospital of China Academy of Chinese Medical Sciences, Beijing 100102, China

## Abstract

Low back pain is a major cause of disability worldwide. Although numerous potential biomarkers for the early diagnosis or treatment of intervertebral disc degeneration (IDD) have been identified subsequent to the development of molecular biology technologies, the mechanisms of IDD remain unknown. Published studies found the unbalance of anabolism and catabolism of annulus fibrosus (AF) played an important role in it. The present study was aimed to identify the potential targets and signaling pathways of IDD, through the combined analysis of differential expression and based on the Gene Expression Omnibus (GEO) dataset from NCBI. PPI Networks Analysis indicated that MMP2 and AGE-RAGE signaling pathway and estrogen signaling pathway may play important roles in initiation and development of IDD. This study forecasted the pathogenesis molecular mechanism of IDD and the potential prognostic and diagnostic biomarkers, but we need to make further molecular biological experiments to confirm our assumptions.

## 1. Introduction

As a major cause of disability worldwide, low back pain (LBP) has increased the social and economic burden significantly [1]. According to the US national health interview survey, 28% of all people had experienced LBP that lasted one day or more during the past three months [2]. Almost half of them suffered from LBP over one year, with a quarter reporting “frequent” pain. In the USA, LBP has become the second frequent reason for visits to the physician and the fifth-ranking cause of admission to hospital and the third most common cause of surgical [3–5]. The total costs of LBP have exceed $100 billion per year in the United States alone according to WHO, which take the number one spot in all healthcare problems [6].

Several published studies have confirmed that LBP was commonly relevant to the intervertebral disc degeneration (IDD) [7, 8]. Although the etiology (ageing, living conditions, biomechanical loading, and genetic factors) [9] and biological process (apoptosis, inflammation) of IDD are difficult to define precisely, there will be similar phenomenon, including the unbalance of anabolism and catabolism of annulus fibrosus (AF) [10], which leads to decreased ability of resisting tension [11] and thus accelerating the process of IDD finally.

Removal of the degenerated or herniated tissue or even the partial or complete replacement of the disc with an artificial substitute is the current popular surgical therapy for IDD [7, 12]. However, this kind of surgery can bring about adjacent disc degeneration or the failure of intervertebral fusion [13, 14]. Therapeutic intervention (recovery the biomechanical and structural properties and restoration the biological behaviors of healthy AF cells) [10] could avoid highly invasive procedures at an early stage of IDD. Now, the multifactorial mechanisms of IDD have achieved substantial advancement, but its initiation and progression are still limited. So it is our key point to find signaling pathways to better understand the cascades of disc degeneration.

Our present study aimed to identify the potential targets and signaling pathways of IDD, through the combined analysis of differential expression and based on the Gene Expression Omnibus (GEO) dataset from NCBI. This may be helpful for the precise treatment of LBP, as well as early diagnosis.

## 2. Materials and Methods

### 2.1. Microarray Date

The gene expression (GSE34095) in the present study was deposited in GEO (http://www.ncbi.nlm.nih.gov/geo/), which was accumulated by Kazezian et al. [7]. GSE34095 datasets were based on Affymetrix GeneChip Human Genome U133A Array. A total of 6 samples were included, containing three nondegenerative disc annulus fibrosus (AF) cells samples and three degenerative disc AF cells samples. Pathway Analysis software system (IPA®, QIAGEN Redwood City) was used for the sequencing process.

### 2.2. Differential Expression Analysis

The GEO2R (http://www.ncbi.nlm.nih.gov/geo/geo2r/) is used to identify differentially expressed genes (DEGs) between degenerative disc AF cells and normal samples. GEO2R is based on R that comes with the GEO databases. Genes with fold change (logFC) >0.5 (upregulated) or <-0.5 (downregulated) and false discovery rate (FDR) adjusted for P<0.1 were considered to be differentially expressed. The Heml software was used to generate the heat maps.

### 2.3. Functional Enrichment Analysis

The differential expression genes were submitted to the Database for Annotation, Visualization, and Integrated Discovery (DAVID, Version6.8, https://david.ncifcrf.gov/) [15] for the analysis of enrichment of gene ontology (GO) terms [16], Kyoto Encyclopedia of Genes and Genomes (KEGG) pathways [17], and Reactome pathways [18]. P<0.05 was considered to indicate a statistically significant difference for the screening of significant GO terms and KEGG pathways.

### 2.4. Protein-Protein Interaction (PPI) Networks Analysis

The differential expression genes were submitted to the Search Tool for the Retrieval of Interacting Genes/Proteins (String, Version 10.0, http://www.string-db.org/) for the analysis of differential protein. PPI score =0.4 was considered that the interacted protein nodes were all transcribed by differentially expressed genes. PPI networks were represented by Cytoscape Software (Version 3.2.0) [19–21].

### 2.5. Subnetworks Analysis

The biological process may interact with multiple genes to play a regulatory role. Generally, those genes perform the same or similar biological function in the subnetworks. The method of MCODE [22] was used to analyze the interacted gene of the significant clustering modules.

## 3. Results

### 3.1. Differentially Expressed Gene in Degenerative Intervertebral Discs Patients

**Figure 1:**
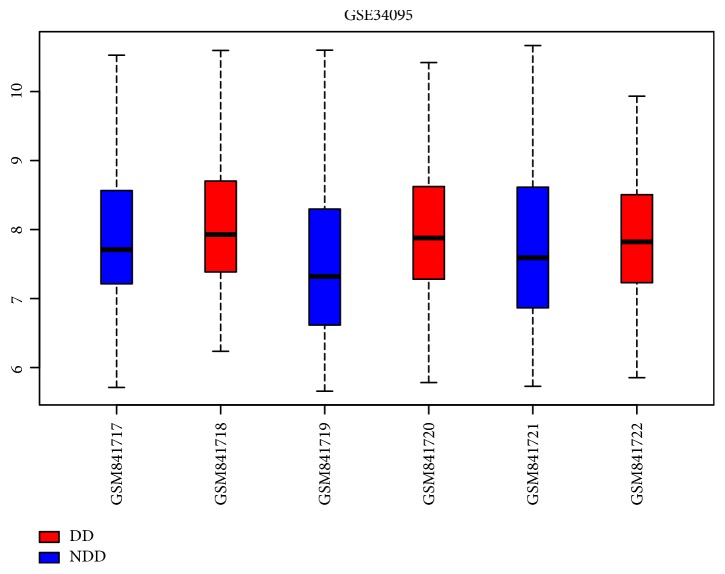
Box plot of GSE34095. Blue: nondegenerative samples; red: degenerative samples.

**Figure 2:**
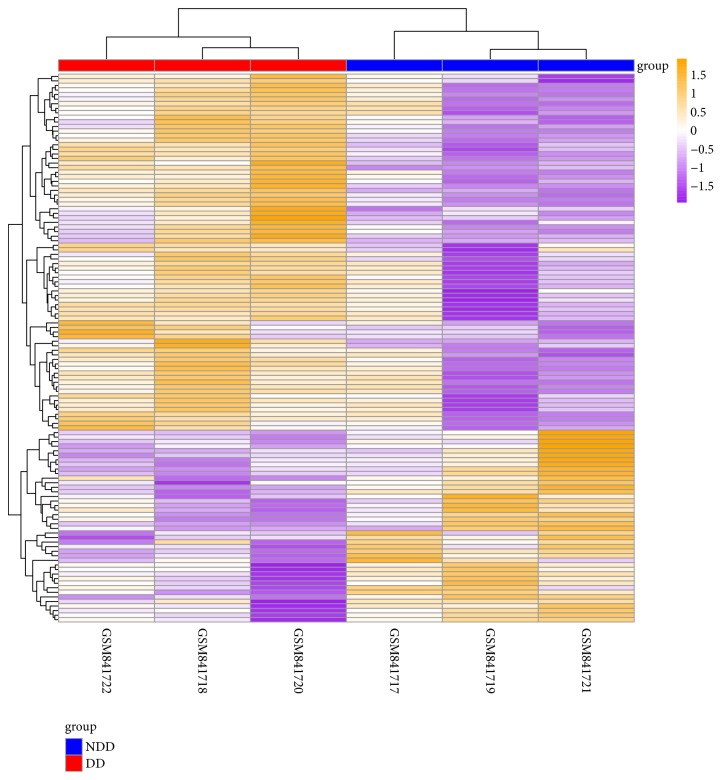
Heat map of differentially expressed in GSE34095 datasets. Yellow: upregulated; purple: downregulated; red: degenerative samples; Blue: nondegenerative samples.

Raw read counts for a total of 22,215 genes were obtained for gene expression analysis. The date normalization of gene expression had no significant difference in 6 samples (Figure 1). Based on the criteria of |logFC| >0.5 and adj. P<0.1, numerous genes were revealed to be differential expression in degenerative samples compared with normal samples (Figure 2). Among them, 42 genes were downregulated and 78 genes were upregulated in degenerative disc AF cells compared to nondegenerative disc AF cells (Figure 2, Table 1).

### 3.2. Functional and Pathway Enrichment Analysis

**Figure 3:**
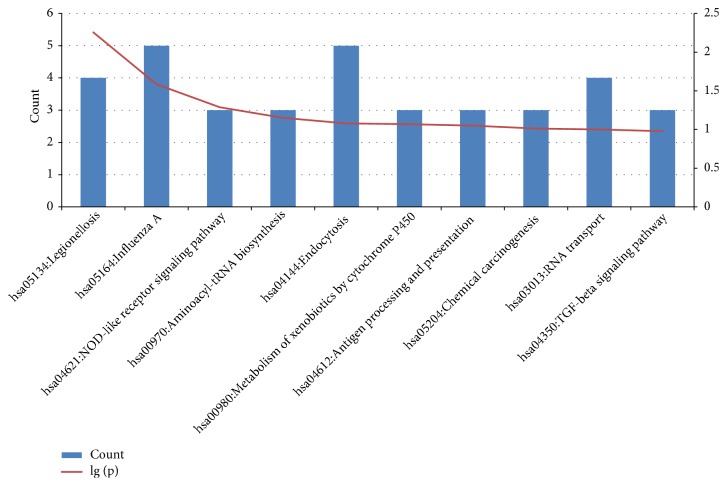
Significantly enriched KEGG pathways of DEGs in degenerative disc AF cells.

To learn more about the function of identified intersection DEGs, functional and pathway enrichment analysis was carried out using DAVID [15]. These DEGs were mainly enriched in pathway associated with endocytosis, influenza A, legionellosis, RNA transport, NOD-like receptor signaling pathway, aminoacyl-tRNA biosynthesis, metabolism of xenobiotics by cytochrome P450, antigen processing and presentation, chemical carcinogenesis, TGF-beta signaling pathway, and AGE-RAGE signaling pathway (Table 2, Figure 3).

### 3.3. PPI Networks Analysis

**Figure 4:**
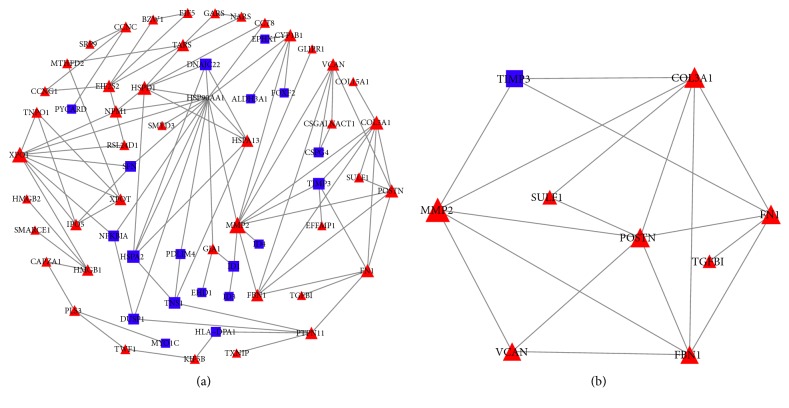
(a) PPI Networks Analysis with Cytoscape for the 120 genes that are differentially expressed between nondegenerative disc AF cells and degenerative disc AF cells samples; (b) module networks of Module A. The red color and triangle in network indicate a gene that is upregulated in degenerative disc AF cells compared to the nondegenerative disc AF cells; blue color indicates the genes that are downregulated in degenerative disc AF cells compared to the nondegenerative disc AF cells samples.

Cytoscape software was used to determine biological relationships among the 120 differentially expressed genes (Figure 4(a)). The top three module networks (Figure 4(b), Table 3), based on Fisher's exact test, were associated with immunological disease, cell signaling, and protein generation pathways. According to the value of degree (degree*⩾*5), we find that the key nodes of the PPI networks included HSP90AA1, COL3A1, MMP2, POSTN, and FN1.

Functional enrichment analysis based on the DAVID identified a number of significantly enriched GO terms and KEGG pathways in module networks DEGs. The significant KEGG pathways of the top three module networks DEGs of degenerative disc AF cells samples are listed in Table 3. As shown in Table 3, KEGG pathways associated with protein processing in endoplasmic reticulum and estrogen signaling pathway were significantly enriched (P<0.05). The top five most significant GOTERM_BP of the module networks DEGs are listed in Table 4. Similar to the KEGG pathways, those GOTERM_BP were mainly involved in the protein processing (assembly, refolding, and stabilization) and degradation (extracellular matrix disassembly).

## 4. Discussion

The tear or partial injury of the intervertebral disc AF is one of the important factors leading to low back pain [23]. With the emerging of IDD studies, numerous potential biomarkers for the early diagnosis or treatment of IDD have been identified subsequent to the development of molecular biology technologies. However, the mechanisms of IDD remain unknown.

In this study, we analyzed the microarray data of degenerative disc AF cells from GEO database under the accession number GSE34095 by GEO2R to obtain DEGs and obtained their enriched GO terms and KEGG pathways. This study aimed to provide important clues for exploring the key genes and associated regulatory network in mechanisms of IDD resulted from AF. Based on the DEGs functional enrichment analysis, potential mechanisms and target gene for disc degeneration caused by AF were suggested as below.

The abnormal expression of MMP2 might cause disc degeneration by accelerating the matrix degradation. According to previous results, a total of 120 DEGs including 42 downregulated and 78 upregulated genes were identified. We selected 10 genes according to the value of degree. Among them, HSP90AA1, MMP2, XPO1, HSPD1, COL3A1, FN1, POSTN, EIF2S2, and TARS were significantly upregulated, and HSPA2 was significantly downregulated in degenerative disc AF cells samples in this study. Recent epidemiologic studies indicated that the key factor for disc degeneration was heredity [24]. The typical character of disc degeneration was the matrix degradation in the early. As an important member of matrix metalloproteinases (MMPs) family, MMP2 plays a critical role in the excessive breakdown of the extracellular matrix (ECM) during disc degeneration [25–27]. The increased expression and activity of MMP2 was responded for degenerative lesions in disc tissue. We reasonably surmise that MMP2 aberrantly expressed plays important roles in initiation and development of IDD.

The activation of AGE-RAGE signaling pathway might cause disc degeneration by accelerating the expression of MMP2. Multiple complexity factors, including age, injury, inflammation, and immunity, activate AGE-RAGE signaling pathway. Based on functional enrichment analysis, two genes (MMP2, FN1) were upregulated in AGE-RAGE signaling pathway. Notably, the two upregulated genes are downstream targets of AGE-RAGE signaling pathway [28]. What is more, AGE-RAGE signaling pathway was found over-expression in degenerative disc AF cells compared with nondegenerative disc AF cells. Taken together, it suggested that multiple complexity factors might lead to IDD by activating AGE-RAGE signaling pathway and then accelerating the expression of downstream targets-MMP2.

Estrogen signaling pathway plays an important role in the process of disc degeneration. Estrogen signaling pathway is ubiquitous in different tissues throughout the body, which participates in many pathological process, such as osteoporosis and osteoarthritis [29]. Recent evidence suggests that 17-beta-estradiol (E_2_) can promote the proliferation of AF cells by activating estrogen beta receptor [30]. Bai et al. [31] found that estrogen could delay the development of ovariectomized rabbit's IDD by reducing the expression of interleukin and MMPs, which could inhibit the degradation of matrix. Kato et al. [32] confirmed that E_2_ could stimulate the expression of COL2A1. However, estrogen secretion will decrease gradually with the increasing of age. It will lead to the activation of estrogen signaling pathway, the expression of inflammatory factors, and the degradation of matrix, which can accelerate the process of IDD.

## 5. Conclusion

Though we identified aberrantly expressed key gene (MMP2) from the GEO database and found the AGE-RAGE signaling pathway and estrogen signaling pathway in degenerative disc AF cells for IDD, which may benefit us in understanding the molecular mechanism of the pathogenesis of IDD and detecting potential prognostic and diagnostic biomarkers, it is still needed that we perform further molecular biological experiments to confirm our assumptions.

## Figures and Tables

**Table 1:** Top 10 differentially expressed genes in datasets GSE34095 according to degree.

Node	HSP90AA1	MMP2	XPO1	HSPD1	COL3A1	FN1	POSTN	EIF2S2	HSPA2	TARS
Description	Up	Up	Up	Up	Up	Up	Up	Up	Down	Up
Degree	15	10	9	7	7	6	6	5	5	5

**Table 2:** Functional and pathway enrichment analysis of target genes in degenerative disc AF cells.

Category	Term	Description	Count	P value
KEGG_PATHWAY	hsa04144	Endocytosis	5	0.0223
KEGG_PATHWAY	hsa05164	Influenza A	5	0.0752
KEGG_PATHWAY	hsa05134	Legionellosis	4	0.0045
KEGG_PATHWAY	hsa03013	RNA transport	4	0.0908
KEGG_PATHWAY	hsa04621	NOD-like receptor signaling pathway	3	0.0456
KEGG_PATHWAY	hsa00970	Aminoacyl-tRNA biosynthesis	3	0.0632
KEGG_PATHWAY	hsa00980	Metabolism of xenobiotics by cytochrome P450	3	0.0771
KEGG_PATHWAY	hsa04612	Antigen processing and presentation	3	0.0807
KEGG_PATHWAY	hsa05204	Chemical carcinogenesis	3	0.0882
KEGG_PATHWAY	hsa04350	TGF-beta signaling pathway	3	0.0958
KEGG_PATHWAY	hsa04933	AGE-RAGE signaling pathway	2	0.0801
REACTOME_PATHWAY	R-HAS-2022870	R-HAS-2022870	3	0.0051
REACTOME_PATHWAY	R-HAS-379716	R-HAS-379716	3	0.0073
REACTOME_PATHWAY	R-HAS-2129379	R-HAS-2129379	3	0.0177
REACTOME_PATHWAY	R-HAS-1442490	R-HAS-1442490	3	0.0466
REACTOME_PATHWAY	R-HAS-300178	R-HAS-300178	3	0.0618
GOTERM_CC_DIRECT	GO:0005737	Cytoplasm	45	0.0001
GOTERM_CC_DIRECT	GO:0070062	Extracellular exosome	37	5.6552E-08
GOTERM_CC_DIRECT	GO:0005634	Nucleus	37	0.0457
GOTERM_MF_DIRECT	GO:0005515	Protein binding	63	0.0005
GOTERM_MF_DIRECT	GO:0005524	ATP binding	14	0.0493
GOTERM_MF_DIRECT	GO:0044822	Poly(A) RNA binding	13	0.0152
GOTERM_MF_DIRECT	GO:009864	Cadherin binding involved in cell-cell adhesion	8	0.0009
GOTERM_MF_DIRECT	GO:0042803	Protein homodimerization activity	8	0.0904
GOTERM_MF_DIRECT	GO:0005201	Extracellular matrix structural constituent	4	0.0053
GOTERM_MF_DIRECT	GO:0051059	NF-kappaB binding	3	0.0109
GOTERM_BP_DIRECT	GO:0007165	Signal transduction	12	0.0457
GOTERM_BP_DIRECT	GO:0045944	Positive regulation of transcription from RNA polymerase II promoter	10	0.0795
GOTERM_BP_DIRECT	GO:0098609	Cell-cell adhesion	9	0.0001

Note: BP, biological process; MF, molecular function; CC, cellular component; GO, gene ontology.

**Table 3:** KEGG pathway enrichment analysis of target genes in the top three module networks.

	Term	Description	Count	P value
Module A	hsa05134	Legionellosis	2	0.0156
	hsa04612	Antigen processing and presentation	2	0.0219
	hsa04915	Estrogen signaling pathway	2	0.0285
	hsa04141	Protein processing in endoplasmic reticulum	2	0.0483

Module B	hsa05134	Legionellosis	2	0.0156
	hsa04612	Antigen processing and presentation	2	0.0219
	hsa04915	Estrogen signaling pathway	2	0.0285
	hsa04141	Protein processing in endoplasmic reticulum	2	0.0483

Module C	hsa05205	Proteoglycans in cancer	3	0.0025
	hsa04512	ECM-receptor interaction	2	0.0373
	hsa05146	Amoebiasis	2	0.0453
	hsa04510	Focal adhesion	2	0.0868

**Table 4:** Top five most significantly enriched GOTERM_BP enrichment analyses of target genes in the top three module networks.

	Term	Description	Count	P value
Module A	GO:0042026	protein refolding	3	2.23E-6
	GO:0009409	response to cold	3	1.34E-5
	GO:0006986	response to unfolded protein	3	1.83E-5
	GO:0009408	response to heat	3	2.40E-5
	GO:0051131	chaperone-mediated protein complex assembly	2	0.0023

Module B	GO:0042026	protein refolding	3	4.46E-6
	GO:0009409	response to cold	3	2.67E-5
	GO:0006986	response to unfolded protein	3	3.65E-5
	GO:0009408	response to heat	3	4.78E-5
	GO:0050821	protein stabilization	3	3.87E-4

Module C	GO:0030198	extracellular matrix organization	4	3.05E-5
	GO:0022617	extracellular matrix disassembly	3	3.00E-4
	GO:0001501	skeletal system development	3	9.70E-4
	GO:0048050	post-embryonic eye morphogenesis	2	0.0014
	GO:0048048	embryonic eye morphogenesis	2	0.0036

## Data Availability

The data used to support the findings of this study are available from the corresponding author upon request.

## Supplementary Materials

Supplementary Materials The highlights are used to describe the findings of this study.
