# Supplementary material for: Integrative Bioinformatics Analysis Reveals Potential Gene Biomarkers and Analysis of Function in Human Degenerative Disc Annulus Fibrosus Cells
Source: Biomed Res Int. 2019 Feb 21;2019:9890279. doi: 10.1155/2019/9890279 (PMC6409044; doi:10.1155/2019/9890279)
Supplement: Supplementary Materials — The highlights are used to describe the findings of this study. [file 9890279.f1.docx]

**Highlights**

- Low Back Pain is relevant to intervertebral disc degeneration;
- Forecasting the potential targets and signalling pathways of disc degeneration;
- The abnormal expression of MMP2 might cause disc degeneration;

• The activation of AGE-RAGE signaling pathway might cause disc degeneration;

• Estrogen signaling pathway plays an important role in the process of disc degeneration.
